# Association of Serum Uric Acid With Retinal Capillary Plexus

**DOI:** 10.3389/fendo.2022.855430

**Published:** 2022-04-12

**Authors:** Kai Yang, Chunmei Li, Keai Shi, Xiaoxuan Zhu, Yunfan Xiao, Binbin Su, Ying Ju, Fan Lu, Jia Qu, Lele Cui, Ming Li

**Affiliations:** Eye Hospital and School of Ophthalmology and Optometry, Wenzhou Medical University, National Clinical Research Center for Ocular Diseases, Wenzhou, China

**Keywords:** serum uric acid, optical coherence tomography angiography, retinal capillary plexus, epidemiology, sex

## Abstract

**Background:**

To determine the association between serum uric acid (SUA) and the retinal capillary plexus (RCP) using optical coherence tomography angiography (OCTA).

**Methods:**

This cross-sectional study evaluated data from August 2019 to January 2020 from participants recruited from the Jidong community (Tangshan, Hebei, China). All participants completed detailed anthropometrical measurements, laboratory tests and comprehensive ophthalmic examinations. We assessed the vessel density in RCP using OCTA. We used multivariable analysis to evaluate the sex-specific association between SUA and RCP after adjusting for confounders.

**Results:**

A total of 2730 participants were included in this study. The mean age of the participants was 44.0 ± 11.6 years, and 1463 (53.6%) were women. The multivariable βs and 95% confidence intervals (CIs) of superficial RCP vessel density in the second through fourth SUA quartiles compared with the lowest SUA quartiles were -0.27 (-0.56 – 0.03), -0.30 (-0.60 – 0.01), and -0.46 (-0.78 – -0.14) (P for trend = 0.007) in men.

**Conclusions:**

Higher SUA levels were significantly associated with lower RCP vessel density in men. Our findings provide evidence for the detrimental effect of high SUA levels on the retinal microvasculature and imply the importance of modulating SUA to prevent the microvascular alternation especially for men.

## Introduction

Serum uric acid (SUA), a product of purine metabolism and its derivatives, has long been associated with various metabolic and cardiovascular diseases (1–4). People with high SUA have an approximately 30% higher rate of cardiovascular disease and all-cause mortality (5). Several studies suggested that higher uric acid level may cause microvascular alterations (6–8), though direct noninvasive *in vivo* observations of microcirculatory alterations are still lacking thus far. The retina is an ideal window for direct noninvasive *in vivo* observation of the microcirculation (9), through which systemic microvascular alterations in persons with high SUA can be indirectly observed. Additionally, sex differences were observed in many conditions about uric acid (10). Thus, it is important to exploring the sex-specific effect of SUA on the retinal microvasculature.

Previously, studies focused on the effect of SUA on retinal arterioles and venules (11, 12) but not on the subtle alteration of retinal capillaries due to the limitations of imaging technology. Recently, optical coherence tomography angiography (OCTA) technology has emerged as a promising new technology for better visualization and quantitative analysis of retinal capillaries (13). Previous studies have reported the wide use of OCTA not only in the assessment of many retinal diseases (14, 15) but also in systemic vascular diseases, such as hypertension (16), stroke (17), coronary artery disease (18), and chronic kidney disease (19). Numerous studies have shown that OCTA-derived vascular metrics, such as the retinal capillary plexus (RCP) vessel density, are useful to evaluate retinal capillary alternations and microvascular pathologic features (20), which may provide new insight into SUA-related effects on the microvasculature.

The relationship between uric acid and retinal microvascular alternations is still unclear. Additionally, close linkages of diabetic retinopathy and hypertensive retinopathy with SUA have been reported previously (21, 22). However, other previous epidemiological reports have failed to demonstrate these relationships (23, 24). Previously, only two relevant studies analyzed the association between higher SUA and retinal vasculature, focusing on retinal arterioles and venules (11, 12). Findings from these studies were inconsistent and contradictory. There is still a lack of studies on the subtle alterations of vessel density in the retinal capillary plexus. In view of this and considering the close linkage between SUA and sex, this community-based study aimed to investigate the sex-specific association of SUA with RCP using OCTA.

## Materials and Methods

### Design and Population of the Study

**Figure 1:**
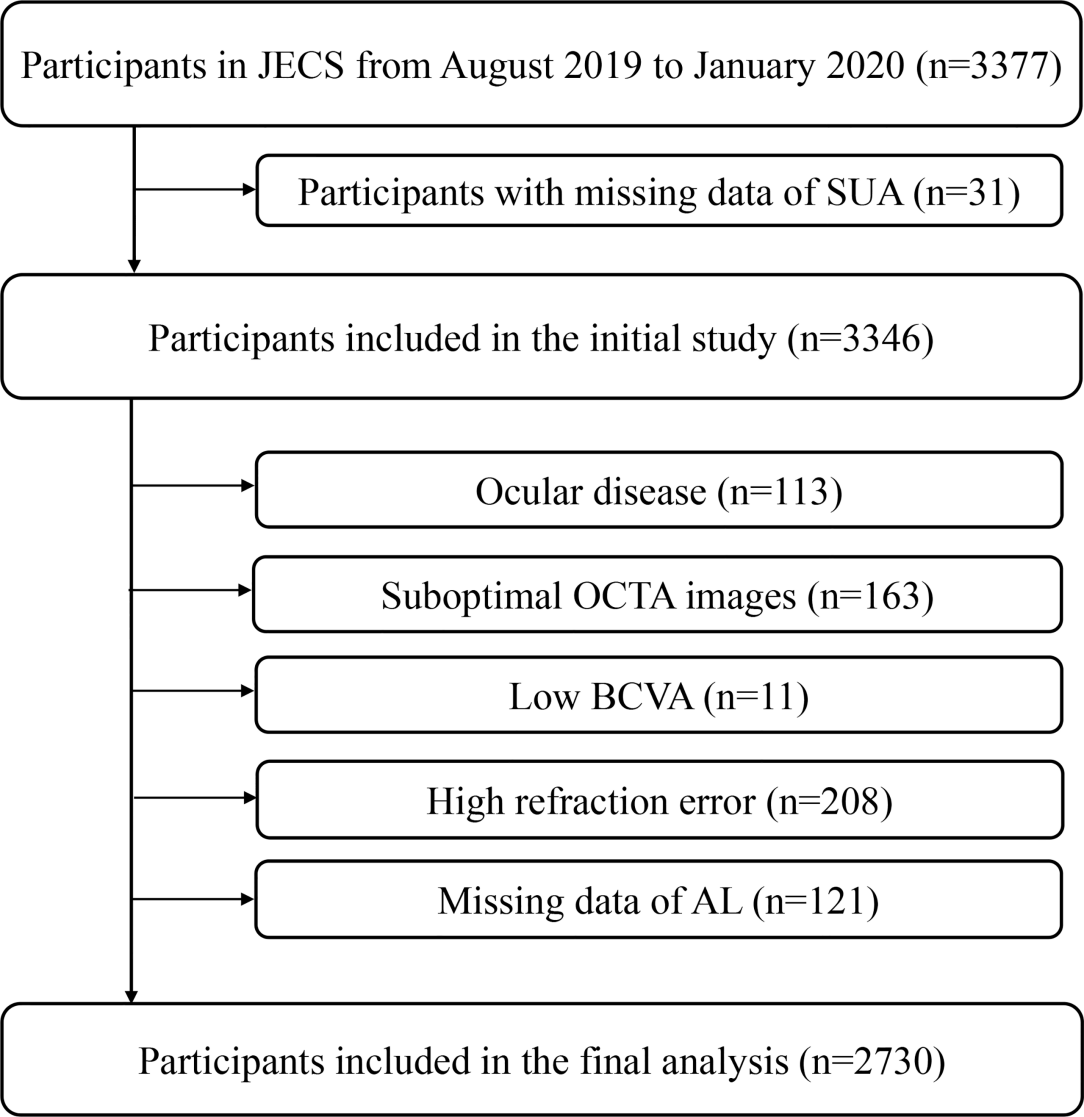
Flow Chart of the Study Participant. JECS, Jidong Eye Cohort Study; OCTA, optical coherence tomography angiography; BCVA, best corrected visual acuity; AL, axial length.

Our study was a substudy of the Jidong Eye Cohort Study (JECS). The data used for this study were obtained from JECS participants. Details of the JECS have been previously described (25). A total of 3377 participants were recruited from Jidong communities (Tangshan, Hebei, China) between August 2019 and January 2020. The following participants were removed from the analyzed sample due to the predetermined exclusion criteria: 31 individuals had missing SUA data; 113 individuals had ocular diseases; 163 individuals had suboptimal OCTA images; 11 individuals had low best corrected visual acuity (BCVA); 208 individuals had refraction above +2.00 or under -6.00 diopters; and 121 individuals had missing axial length data (**Figure 1**).

This study was approved by the Ethics Committee of the Staff Hospital of Jidong oil-field of Chinese National Petroleum (China national petroleum corporation Jidong oilfield branch staff hospital approval document of the medical ethics committee, 2018 YILUNZI 1) and followed the Declaration of Helsinki guidelines. We obtained written informed consent forms from all participants.

### Assessment of General Variable

In this study, basic information on the participants was obtained using clinical examinations, laboratory tests, and standardized questionnaires for demographic characteristics, current smoking status, current alcohol consumption status, and medical history (26, 27). The average monthly income levels were divided into “< ¥3,000” and “≥ ¥3,000”. The education levels were categorized as “illiteracy, primary or middle school” and “college graduate or above”. In this study, diabetes was determined by either fasting blood glucose (FBG) ≥ 7.0 mmol/L, self-reported diabetes history, or current antidiabetic medication use; while hypertension was defined as either systolic blood pressure ≥ 140 mmHg, diastolic blood pressure ≥ 90 mmHg, self-reported hypertension history, or current use of antihypertensive medications. Dyslipidemia was defined as either low-density lipoprotein (LDL-C) ≥ 3.3 mmol/L, high-density lipoprotein (HDL-C) < 1.04 mmol/L, total cholesterol (TC) ≥ 5.17 mmol/L, triglyceride (TG) ≥ 1.7 mmol/L, current use of lipid-lowering medications, or self-reported history of dyslipidemia. We calculated the estimated glomerular filtration rate (eGFR) using the Chronic Kidney Disease Epidemiology Collaboration creatinine equation and adjusted it by a coefficient of 1.1 for the Asian population (28, 29).

### Assessment of Serum Uric Acid

Fasting elbow venous blood samples were obtained in the morning after refraining from food and drinking for at least 8 hours and they were stored in vacuum tubes containing ethylenediaminetetraacetic acid. SUA levels were measured using an autoanalyzer (Hitachi, Tokyo, Japan) with the uricase-peroxidase method in the Central Laboratory of Jidong Oil-Field Hospital (25, 30). Participants in this study were stratified into quartiles based on sex-specific SUA levels (male, Q1: ≤ 335 μmol/L, Q2: 336 - 385 μmol/L, Q3: 386 - 443 μmol/L, Q4: ≥ 444 μmol/L; female, Q1: ≤ 244 μmol/L, Q2: 245 - 278 μmol/L, Q3: 279 - 322 μmol/L, Q4: ≥ 323 μmol/L). Hyperuricemia was defined as an SUA level ≥ 360 µmol/L (6.0 mg/dL) in women and ≥ 420 µmol/L (7.0 mg/dL) in men (31).

### Ophthalmic Examination

Participants in our study underwent a comprehensive eye examination including best-corrected visual acuity (BCVA) by a standard logarithmic visual acuity chart and refractive status by an autorefractometer (KR800; Topcon; Tokyo, Japan). Refraction was calculated as the spherical equivalent (spherical value and half of the cylindrical value). Measurements of axial length (AL) were obtained using a Lenstar 900 (Haag-Streit; Koeniz, Switzerland). Digital fundus photographs were obtained using a 45° nonmydriatic fundus camera (CR2AF; Canon; Tokyo, Japan). All examination results were reviewed by at least two ophthalmologists. Participants with ocular disease, low BCVA (worse than 0.5 logMAR), or high refraction error (spherical equivalent < -6.00 D/spherical equivalent > +2.00 D) were excluded from the analyses.

### Assessment of OCTA

We obtained OCTA images using a spectral-domain OCTA device (RTVue XR Avanti with AngioVue; Optovue; Fremont, CA, USA). OCTA images were acquired using a 3 × 3 mm² scan with a scan density of 304×304 A-scans centered on the macula. The superficial (between the internal limiting membrane and inner plexiform layer) and deep (between the inner plexiform layer and outer plexiform layer) RCPs were automatically segmented according to the built-in software (AngioAnalytics, version 2017.1.0.155) (25). An algorithm incorporated into the device (optovue, Inc) automatically removes the 3D projection artifacts to reduce the motion artifacts and increase the image quality (32). Superficial and deep OCTA images were exported and then corrected using Bennett’s formula with AL magnification (33). The vessel density (the proportion of the flow signals) of the superficial and deep RCP was automatically generated by a custom algorithm operating on MATLAB (version 2017b; MathWorks, Inc.; Natick, MA, USA). Details of the processing and analysis methods have been previously described (33, 34). The processing algorithm based on a macula map was as follows: two concentric circles (0.6 and 2.5 mm diameters) divided into five quadrants (parafovea, superior, inferior, nasal, and temporal). In this report, the quadrant of the parafovea served as a representative of the average RCP due to the special structure of the foveal area, and the other quadrants were also presented.

Only OCTA scans with a signal strength index ≥ 7 were included for quantitative analysis and images with defects were excluded from the study. We used the measurement of the right eye if it was available, whereas the left eye was only analyzed in the absence of eligible scans for the right eye.

### Statistical Analysis

We expressed continuous variables as the means and standard deviations (SD) and categorical valuables as frequencies and percentages. We examined the differences between the different SUA quartile groups using a one-way ANCOVA test for normally distributed continuous variables and chi-square tests or Fisher’s exact test for categorical valuables.

We used multivariable generalized linear models to estimate the association between the SUA quartile and the vessel density in the RCP. We tested for trends by considering the SUA quartile as a continuous ordinal variable. We adjusted all multivariable generalized linear models for age, current drinking status, hypertension, diabetes, body mass index (BMI), TC, TG, HDL-C, LDL-C, and eGFR. Moreover, associations of a 1 standard deviation change in SUA and the presence of hyperuricemia with RCP vessel density were also tested, and we examined whether the associations between SUA and RCP differed by sex by including interaction terms in the adjusted models.

Associations were measured as βs and 95% confidence intervals (CIs). For all analyses, statistical significance was considered as a 2-tailed P value < 0.05. All statistical analyses were undertaken using SAS software (version 9.4; SAS Institute Inc., Cary, NC, USA).

## Results

### Baseline Characteristics

A total of 2730 participants from Jidong communities were eventually included in the analyses. The mean age of the included participants was 44.0 ± 11.6 years, and there were 1463 (53.6%) women. Baseline characteristics by different quartiles of serum SUA levels are summarized in **Table 1**. Participants with higher SUA levels were younger (P = 0.006), more likely to be drinkers (p = 0.01), have a higher BMI (P < 0.001), have a lower eGFR (P < 0.001), and have more prevalent hypertension (P < 0.001) and dyslipidemia (P < 0.001).

**Table 1:** Baseline characteristics of participants by serum uric acid quartiles.

Characteristics	Total	SUA				P	P for Trend
	(n=2730)	Q1 (n=692)	Q2 (n=682)	Q3 (n=679)	Q4 (n=677)		
Age, y, mean (SD)	44.0 (11.6)	45.1 (11.5)	43.9 (11.1)	43.6 (11.8)	43.4 (12.0)	0.03	0.006
Sex, n (%)						0.99	0.96
Male	1267 (46.4)	320 (46.2)	319 (46.8)	312 (46.0)	316 (46.7)		
Female	1463 (53.6)	372 (53.8)	363 (53.2)	367 (54.1)	361 (53.3)		
Educational level, n (%)						0.69	0.77
lliteracy/Primary/Middle School	849 (31.1)	225 (32.5)	205 (30.1)	204 (30.0)	215 (31.8)		
College/University	1881 (68.9)	467 (67.5)	477 (69.9)	475 (70.0)	462 (68.2)		
Income, n (%)						0.31	0.94
< ¥3,000	216 (7.9)	62 (9.0)	43 (6.3)	55 (8.1)	56 (8.3)		
≥ ¥3,000	2514 (92.1)	630 (91.0)	639 (93.7)	624 (91.9)	621 (91.7)		
Current Smoking, n (%)	532 (19.6)	139 (20.3)	116 (17.1)	132 (19.5)	145 (21.6)	0.20	0.36
Current Drinking, n (%)	377 (14.0)	83 (12.2)	85 (12.5)	98 (14.5)	111 (16.6)	0.07	0.01
Hypertension, n (%)	716 (26.2)	157 (22.7)	157 (23.0)	170 (25.0)	232 (34.3)	< 0.001	< 0.001
Diabetes, n (%)	229 (8.4)	71 (10.3)	55 (8.1)	47 (6.9)	56 (8.3)	0.16	0.13
Dyslipidemia, n (%)	1831 (67.1)	386 (55.8)	429 (62.9)	461 (67.9)	555 (82.0)	< 0.001	< 0.001
SBP, mmHg, mean (SD)	123.7 (16.8)	122.0 (17.1)	121.6 (16.2)	123.3 (15.9)	127.9 (17.1)	< 0.001	< 0.001
DBP, mmHg, mean (SD)	78.8 (12.6)	77.2 (12.6)	77.5 (12.2)	78.9 (12.2)	81.5 (12.7)	< 0.001	< 0.001
FBG, mmol/L, mean (SD)	5.5 (1.4)	5.6 (1.7)	5.5 (1.4)	5.4 (1.2)	5.5 (1.1)	0.13	0.49
BMI, kg/m^2^, mean (SD)	24.4 (3.5)	23.3 (3.2)	2.8 (3.2)	24.5 (3.4)	26.0 (3.6)	< 0.001	< 0.001
TC, mmol/L, mean (SD)	5.0 (1.0)	4.8 (0.9)	4.9 (0.9)	5.0 (0.9)	5.3 (1.0)	< 0.001	< 0.001
Triglycerides, mmol/L, mean (SD)	1.7 (1.4)	1.4 (1.0)	1.6 (1.3)	1.7 (1.3)	2.2 (1.8)	< 0.001	< 0.001
HDL-C, mmol/L, mean (SD)	1.2 (0.3)	1.3 (0.3)	1.2 (0.3)	1.2 (0.2)	1.2 (0.2)	< 0.001	< 0.001
LDL-C, mmol/L, mean (SD)	2.1 (0.8)	1.9 (0.7)	2.0 (0.7)	2.1 (0.7)	2.3 (0.8)	< 0.001	< 0.001
AL, mm, mean (SD)	24.1 (1.2)	24.0 (1.2)	24.1 (1.2)	24.1 (1.3)	24.1 (1.2)	0.08	0.07
OCT signal index, mean (SD)	8.5 (0.7)	8.5 (0.7)	8.5 (0.6)	8.4 (0.7)	8.5 (0.6)	0.06	0.14
eGFR, ml/min*1.73m^2^, mean (SD)	114.7 (14.9)	117.2 (12.7)	116.0 (13.7)	114.5 (14.6)	111.2 (17.6)	< 0.001	< 0.001
Serum uric acid, umol/L, mean (SD)	336.9 (91.2)	252.9 (47.8)	308.9 (52.1)	351.5 (58.8)	436.2 (83.3)	< 0.001	< 0.001
Serum uric acid, range	129 - 758	129 - 335	245 - 385	279 - 443	323 - 758		

Data were presented as number (percentage) for category variables and mean (SD) for continuous variables.

P for trend tested by considering the SUA quartile as continuous ordinal variables.

The quartiles of SUA levels were calculated by sex respectively. Q1, quartile 1 (n=692): male ≤ 335 umol/L and female ≤ 244 umol/L; Q2, quartile 2 (n=682): male 336 -385 umol/L and female 245 – 278 umol/L; Q3, quartile 3 (n=679): male 386 – 443 umol/L and female 279 - 332 umol/L; Q4, quartile 4 (n=677): male ≥ 444 umol/L and female ≥ 323 umol/L.

SUA, Serum Uric Acid; SBP, systolic blood pressure; DBP, diastolic blood pressure; FBG, fasting blood glucose; BMI, body mass index; TC, total cholesterol; HDL-C, high density liptein cholesterol; LDL-C, low density lipoprotein cholesterol; AL, axial length; OCT, Optical coherence tomography; eGFR, estimated glomerular filtration rate.

### RCP Vessel Density in Sex-Specific SUA Quartile Groups

**Figure 2:**
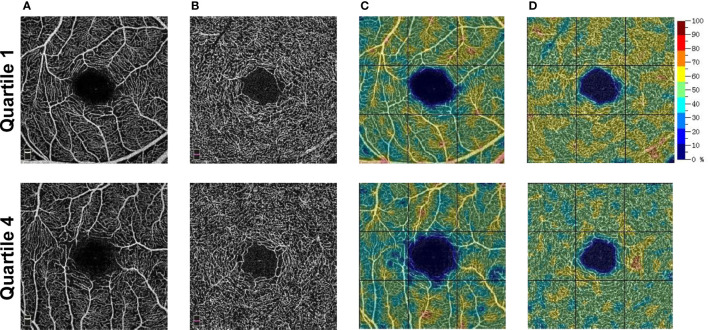
Representative OCTA images of male participants. **(A)** The superficial OCT angiograms. **(B)** The deep OCT angiograms. **(C)** The color-coded superficial RCP vessel density maps. **(D)** The color-coded deep RCP vessel density maps. Quartile 1, SUA level ≤335 umol/L; Quartile 4, SUA level ≥444 umol/L. OCTA, optical coherence tomography angiography; OCT, optical coherence tomography; SUA, serum uric acid; RCP, retinal capillary plexus.

**Table 2** shows the RCP characteristics in different SUA quartile groups on the baseline survey. For the male participants stratified into quartiles, the vessel density of the superficial RCP decreased from Q1 to Q4 from 51.1% to 50.6% in the parafovea. For the vessel density in other quadrants of RCP, see **Table 2**. However, we did not observe a significant difference in deep RCP in men or superficial and deep RCP in women. **Figure 2** shows the representative OCTA images of male participants in Q1 and Q4.

**Table 2:** Ocular characteristics of participants by serum uric acid quartiles in males and females.

Characteristics	Total	SUA				P	P for Trend
		Q1	Q2	Q3	Q4		
**Male**	**n=1267**	**n=320**	**n=319**	**n=312**	**n=316**		
Superficial RCP, %, mean (SD)							
Parafovea	50.8 (1.9)	51.1 (2.0)	50.8 (1.8)	50.7 (2.0)	50.6 (1.7)	0.02	0.003
Inferior	50.5 (2.0)	50.8 (2.1)	50.5 (1.9)	50.5 (2.0)	50.3 (1.8)	0.07	0.01
Nasal	50.7 (2.0)	51.0 (2.1)	50.7 (2.0)	50.6 (2.1)	50.4 (1.9)	0.008	0.001
Superior	50.8 (2.0)	51.1 (2.2)	50.8 (1.9)	50.7 (2.0)	50.7 (1.8)	0.12	0.03
Temporal	51.2 (2.1)	51.5 (2.2)	51.2 (2.1)	51.1 (2.2)	51.0 (1.9)	0.02	0.001
Deep RCP, %, mean (SD)							
Parafovea	54.0 (2.9)	54.2 (3.0)	54.0 (3.0)	54.0 (3.0)	53.8 (2.6)	0.53	0.15
Inferior	54.0 (3.0)	54.2 (3.1)	54.0 (3.1)	54.0 (3.1)	53.9 (2.7)	0.47	0.14
Nasal	53.8 (2.9)	53.9 (3.1)	53.8 (3.0)	53.8 (2.9)	53.5 (2.7)	0.43	0.12
Superior	54.2 (3.2)	54.4 (3.3)	54.2 (3.2)	54.2 (3.3)	54.2 (2.8)	0.72	0.41
Temporal	53.9 (3.0)	54.1 (3.0)	54.0 (3.1)	53.9 (3.1)	53.7 (2.8)	0.39	0.08
**Female**	**n=1463**	**n=372**	**n=363**	**n=367**	**n=361**		
Superficial RCP, %, mean (SD)							
Parafovea	50.3 (1.9)	50.4 (1.9)	50.2 (2.0)	50.3 (1.9)	50.5 (2.0)	0.12	0.33
Inferior	50.1 (2.0)	50.2 (1.9)	50.0 (2.0)	50.1 (2.0)	50.3 (2.0)	0.18	0.40
Nasal	50.1 (2.0)	50.2 (2.0)	50.0 (2.1)	50.0 (2.0)	50.3 (2.0)	0.10	0.57
Superior	50.4 (2.0)	50.3 (2.1)	50.3 (2.1)	50.3 (1.9)	50.5 (2.0)	0.41	0.22
Temporal	50.7 (2.2)	50.8 (2.1)	50.6 (2.2)	50.6 (2.1)	51.0 (2.2)	0.04	0.30
Deep RCP, %, mean (SD)							
Parafovea	54.2 (2.8)	54.3 (2.9)	54.1 (2.8)	54.2 (2.7)	54.2 (2.7)	0.70	0.86
Inferior	54.3 (3.0)	54.4 (3.2)	54.2 (3.0)	54.3 (2.9)	54.5 (2.9)	0.52	0.85
Nasal	53.9 (2.9)	53.9 (3.1)	53.7 (2.8)	53.8 (2.7)	53.9 (2.8)	0.83	0.98
Superior	54.6 (3.0)	54.6 (3.0)	54.5 (3.0)	54.6 (2.9)	54.5 (2.9)	0.93	0.80
Temporal	54.0 (2.9)	54.2 (2.9)	53.9 (2.9)	53.9 (2.8)	54.1 (2.8)	0.38	0.53

Data were presented as mean (SD)SD for continuous variables.

P for trend tested by considering the SUA quartile as continuous ordinal variables.

The quartiles of SUA levels were calculated by sex respectively. In males: Q1, quartile 1 (n=320): ≤ 335 umol/L; Q2, quartile 2 (n=319): 336 - 385 umol/L; Q3, quartile 3 (n=312): 386 - 443 umol/L; Q4, quartile 4 (n=316): ≥ 444 umol/L. In females: Q1, quartile 1 (n=372): ≤ 244 umol/L; Q2, quartile 2 (n=363): 245 - 278 umol/L; Q3, quartile 3 (n=367): 279 - 322 umol/L; Q4, quartile 4 (n=361): ≥ 323 umol/L; n, number.

SUA, Serum Uric Acid; RCP, retinal capillary plexus.

### Association Between SUA and RCP by Sex

We show the relationships between SUA quartile and RCP vessel density for men in **Table 3**. Male participants in the highest SUA quartile had a significant decrease in vessel density in the superficial RCP, even after adjusting for potential confounding factors. Compared to the lowest quartile, the multivariable βs and 95% Cis for the male participants in the second through fourth SUA quartiles were -0.27 (-0.56 – 0.03), -0.30 (-0.60 – 0.01), and -0.46 (-0.78 – -0.14) (P for trend = 0.007) in the parafovea. The βs in other quadrants of RCP are also shown. However, for deep RCP vessel density, we observed no significant association.

**Table 3:** Association between serum uric acid and retinal capillary plexus in men.

Characteristics	SUA				P for Trend
	Q1 (n=320)	Q2 (n=319)	Q3 (n=312)	Q4 (n=316)	
Superficial RCP					
Parafovea	Referent	-0.27 (-0.56 – 0.03)	-0.30 (-0.60 – 0.01)	-0.46 (-0.78 – -0.14)	0.007
Inferior	Referent	-0.24 (-0.55 – 0.06)	-0.24 (-0.56 – 0.07)	-0.41 (-0.74 – -0.08)	0.02
Nasal	Referent	-0.30 (-0.62 – 0.01)	-0.29 (-0.62 – 0.03)	-0.55 (-0.89 – -0.21)	0.003
Superior	Referent	-0.27 (-0.58 – 0.05)	-0.30 (-0.62 – 0.02)	-0.36 (-0.70 – -0.03)	0.04
Temporal	Referent	-0.25 (-0.57 – 0.08)	-0.36 (-0.70 – -0.02)	-0.51 (-0.86 – -0.16)	0.004
Deep RCP					
Parafovea	Referent	-0.19 (-0.64 – 0.26)	-0.18 (-0.65 – 0.28)	-0.31 (-0.79 – 0.17)	0.25
Inferior	Referent	-0.24 (-0.71 – 0.23)	-0.21 (-0.69 – 0.27)	-0.41 (-0.91 – 0.10)	0.15
Nasal	Referent	-0.08 (-0.54 – 0.38)	-0.10 (-0.57 – 0.37)	-0.31 (-0.80 – 0.19)	0.24
Superior	Referent	-0.31 (-0.81 – 0.19)	-0.26 (-0.76 – 0.25)	-0.21 (-0.74 – 0.32)	0.50
Temporal	Referent	-0.12 (-0.58 – 0.35)	-0.17 (-0.65 – 0.31)	-0.31 (-0.81 – 0.18)	0.22

Adjusted for age, current drinking status, hypertension, diabetes, BMI, TC, TG, HDL-C, LDL-C, and eGFR.

P for trend tested with generalized linear models by considering the SUA quartile as continuous ordinal variables.

Q1, quartile 1 (n=320): ≤ 335 umol/L; Q2, quartile 2 (n=319): 336 - 385 umol/L; Q3, quartile 3 (n=312): 386 - 443 umol/L; Q4, quartile 4 (n=316): ≥ 444 umol/L.

SUA, Serum Uric Acid; RCP, retinal capillary plexus.

We show the relationships between SUA quartile and RCP vessel density for women in **Table 4**. No significant associations were found between SUA levels and RCP vessel density in women.

**Table 4:** Association between serum uric acid and retinal capillary plexus in women.

Characteristics	SUA				P for Trend
	Q1 (n=372)	Q2 (n=363)	Q3 (n=367)	Q4 (n=361)	
Superficial RCP					
Parafovea	Referent	-0.24 (-0.52 – 0.04)	-0.24 (-0.52 – 0.05)	-0.16 (-0.46 – 0.14)	0.29
Inferior	Referent	-0.25 (-0.54 – 0.04)	-0.24 (-0.53 – 0.05)	-0.21 (-0.52 – 0.11)	0.20
Nasal	Referent	-0.31 (-0.60 – -0.01)	-0.28 (-0.58 – 0.01)	-0.19 (-0.51 – 0.13)	0.24
Superior	Referent	-0.12 (-0.41 – 0.18)	-0.10 (-0.40 – 0.19)	-0.10 (-0.41 – 0.22)	0.56
Temporal	Referent	-0.30 (-0.61 – 0.02)	-0.31 (-0.63 – 0.00)	-0.15 (-0.49 – 0.19)	0.34
Deep RCP					
Parafovea	Referent	-0.29 (-0.70 – 0.11)	-0.14 (-0.55 – 0.27)	-0.11 (-0.55 – 0.33)	0.76
Inferior	Referent	-0.34 (-0.77 – 0.10)	-0.13 (-0.57 – 0.31)	-0.02 (-0.49 – 0.45)	0.90
Nasal	Referent	-0.26 (-0.68 – 0.16)	-0.10 (-0.52 – 0.32)	-0.10 (-0.56 – 0.35)	0.81
Superior	Referent	-0.18 (-0.62 – 0.25)	-0.00 (-0.44 – 0.44)	-0.07 (-0.54 – 0.39)	0.96
Temporal	Referent	-0.40 (-0.82 – 0.02)	-0.32 (-0.74 – 0.10)	-0.25 (-0.71 – 0.20)	0.32

Adjusted for age, current drinking status, hypertension, diabetes, BMI, TC, TG, HDL-C, LDL-C, and eGFR.

P for trend tested with generalized linear models by considering the SUA quartile as continuous ordinal variables.

Q1, quartile 1 (n=372): ≤ 244 umol/L; Q2, quartile 2 (n=363): 245 - 278 umol/L; Q3, quartile 3 (n=367): 279 - 322 umol/L; Q4, quartile 4 (n=361): ≥ 323 umol/L.

SUA, Serum Uric Acid; RCP, retinal capillary plexus.

We observed a significant moderating effect of sex on the association between SUA and RCP. SUA levels were relatively more strongly associated with low superficial RCP vessel density (parafovea, interaction P=0.02) in men than in women (**Table 5**).

**Table 5:** Impact of serum uric acid on retinal capillary plexus according to sex.

Characteristics	Sex		P for
	Male (n=1267)	Female (n=1463)	interaction
Superficial RCP			
Parafovea	-0.14 (-0.24 – -0.04)	-0.05 (-0.15 – 0.04)	0.02
Inferior	-0.12 (-0.23 – -0.02)	-0.06 (-0.16 – 0.03)	0.07
Nasal	-0.16 (-0.27 – -0.05)	-0.06 (-0.16 – 0.04)	0.02
Superior	-0.11 (-0.22 – -0.01)	-0.03 (-0.13 – 0.07)	0.05
Temporal	-0.16 (-0.28 – -0.05)	-0.05 (-0.16 – 0.05)	0.02
Deep RCP			
Parafovea	-0.09 (-0.25 – 0.06)	-0.02 (-0.16 – 0.12)	0.13
Inferior	-0.12 (-0.28 – 0.04)	0.01 (-0.14 – 0.16)	0.08
Nasal	-0.09 (-0.25 – 0.06)	-0.02 (-0.16 – 0.13)	0.10
Superior	-0.06 (-0.23 – 0.11)	-0.00 (-0.15 – 0.14)	0.25
Temporal	-0.10 (-0.26 – 0.06)	-0.07 (-0.22 – 0.07)	0.23

Adjusted for age, current drinking status, hypertension, diabetes, BMI, TC, TG, HDL-C, LDL-C, and eGFR.

The SUA quartile were considered as continuous ordinal variables.

Interaction effect was calculated from models that included interaction terms of factor x continuous SUA quartiles, and were adjusted for age, current drinking status, hypertension, diabetes, BMI, TC, TG, HDL-C, LDL-C, and eGFR.

RCP, retinal capillary plexus.

### Sensitivity Analysis

We show the adjusted associations between hyperuricemia and RCP in **Table 6**. After adjusting for the confounders mentioned above, we found significant associations between hyperuricemia and superficial RCP vessel density (parafovea: β = -0.24, 95% CI, -0.47 to -0.01) in men but not in women. However, no significant association was found between hyperuricemia and deep RCP in either sex. Moreover, associations between 1 standard deviation altered in SUA and the RCP vessel density were also tested, as shown in **Table 7**. We observed similar associations.

**Table 6:** Association between hyperuricemia and retinal capillary plexus.

Characteristics	Sex		P for
	male (n=1267)	female (n=1463)	interaction
Superficial RCP			
Parafovea	-0.24 (-0.47 – -0.01)	0.03 (-0.29 – 0.35)	0.02
Inferior	-0.21 (-0.45 – 0.04)	0.02 (-0.31 – 0.35)	0.04
Nasal	-0.30 (-0.55 – -0.05)	0.13 (-0.20 – 0.47)	0.004
Superior	-0.15 (-0.40 – 0.10)	-0.07 (-0.40 – 0.27)	0.20
Temporal	-0.32 (-0.58 – -0.06)	0.01 (-0.35 – 0.36)	0.02
Deep RCP			
Parafovea	-0.11 (-0.46 – 0.25)	0.27 (-0.19 – 0.73)	0.04
Inferior	-0.13 (-0.50 – 0.24)	0.36 (-0.14 – 0.85)	0.04
Nasal	-0.13 (-0.50 – 0.23)	0.34 (-0.14 – 0.81)	0.02
Superior	-0.02 (-0.41 – 0.37)	0.18 (-0.31 – 0.68)	0.22
Temporal	-0.15 (-0.52 – 0.22)	0.21 (-0.27 – 0.69)	0.05

Adjusted for age, current drinking status, hypertension, diabetes, BMI, TC, TG, HDL-C, LDL-C, and eGFR.

Hyperuricemia was considered as SUA ≥ 420 µmol/L in males and ≥ 360 µmol/L in females.

Interaction effect was calculated from models that included interaction terms of factor x hyperuricemia, and were adjusted for age, current drinking status, hypertension, diabetes, BMI, TC, TG, HDL-C, LDL-C, and eGFR.

RCP, retinal capillary plexus.

**Table 7:** Association between continuous serum uric acid and retinal capillary plexus.

Characteristics	Sex		P for
	Male (n=1267)	Female (n=1463)	interaction
Superficial RCP, per 1 SD increase.			
Parafovea	-0.19 (-0.32 – -0.06)	-0.04 (-0.19 – 0.11)	0.01
Inferior	-0.17 (-0.30 – -0.03)	-0.05 (-0.21 – 0.10)	0.03
Nasal	-0.21 (-0.35 – -0.08)	-0.04 (-0.19 – 0.12)	0.007
Superior	-0.17 (-0.30 – -0.04)	-0.02 (-0.18 – 0.13)	0.02
Temporal	-0.22 (-0.36 – -0.08)	-0.06 (-0.23 – 0.11)	0.01
Deep RCP, per 1 SD increase.			
Parafovea	-0.14 (-0.33 – 0.05)	0.09 (-0.13 – 0.31)	0.02
Inferior	-0.17 (-0.37 – 0.03)	0.16 (-0.07 – 0.39)	0.01
Nasal	-0.16 (-0.35 – 0.04)	0.11 (-0.10 – 0.33)	0.03
Superior	-0.10 (-0.31 – 0.11)	0.07 (-0.16 – 0.30)	0.08
Temporal	-0.12 (-0.32 – 0.08)	0.02 (-0.20 – 0.25)	0.05

Adjusted for age, current drinking status, hypertension, diabetes, BMI, TC, TG, HDL-C, LDL-C, and eGFR.

The SUA alternations were defined as per each SD increase.

Interaction effect was calculated from models that included interaction terms of factor x SUA, and were adjusted for age, current drinking status, hypertension, diabetes, BMI, TC, TG, HDL-C, LDL-C, and eGFR.

RCP, retinal capillary plexus.

## Discussion

In this OCTA study, we evaluated the detrimental effect of high SUA levels on RCP in a relatively large community-based population. The multivariable generalized linear model analysis showed that the male participants with SUA levels in the highest quartile were significantly associated with lower RCP vessel density after adjusting for confounders.

Collectively, the studies to date have not shown a clear relationship between SUA and retinal vascular metrics (11, 12). In our study, we showed that higher SUA levels are associated with lower vessel density only in men. In supporting of our findings, YuanZhi et al. (11) previously showed an association between an elevated SUA and a smaller retinal arteriolar caliber and larger retinal venular caliber, and the associations were more pronounced in men than in women. However, a study in a Chinese coastal population showed that a higher SUA was associated with a larger retinal arteriolar caliber and larger retinal venular caliber only in women (12). This pattern of inconsistent findings may be explained by different SUA levels, different characteristics of the participants, and different imaging methods.

As reported in previous studies, the loss of blood flow or hypoperfusion in retinal vasculature leads to several ocular diseases, such as hypertensive retinopathy (22) and diabetic retinopathy (35, 36). Findings from our studies may help explain why people with higher SUA levels are more likely to develop ocular disorders and visual impairment (21, 37). However, it is important to point out that higher SUA levels are associated with only a small reduction in RCP vessel density, which may not reach clinical relevance, although it was statistically significant. Potentially, the reduced RCP in participants with higher SUA may be explained by the underlying physiological mechanism by which uric acid induces endothelial dysfunction (38, 39) and stimulates endothelial cell proliferation (40) by reducing nitric oxide and activating the renin-angiotensin system (41) and eventually leading to microvascular damage (42).

In our study, significant sex differences in SUA levels and their association with RCP vessel density were observed. We found that SUA levels were associated with RCP vessel density in men but not in women. It is important to point out that the man sex is a significant risk factor for high SUA levels, as man are up to four times more likely to be affected (43). Our findings suggest that retinal microvasculature in man are more possible to be affected by high SUA levels than women. Our study emphasized the importance of modulating SUA for men. The sex differences in this study may be attributed to menopause and estrogen (10), as we observed a significant fluctuation in SUA levels around the period of menopause in women. The studies mentioned above showed inconsistent findings (11, 12). In addition, a study in Japan reported an association between SUA levels and diabetic retinopathy only in men (44). Nevertheless, other studies previously showed that an increase in SUA can be more detrimental in women (10). The discrepancy may be attributed to age, SUA levels, hormone levels, and other cohort differences. Further research is still required.

Surprisingly, we observed that SUA levels were associated with vessel density only in the superficial RCP but not in the deep RCP. OCTA imaging in the deep retinal OCT angiogram, in contrast to superficial imaging, is more likely to be affected by motion artifacts, and the quantification of flow density in deep RCP is more challenging. Previous studies have shown that the repeatability of deep retinal OCT angiograms is weaker than that of superficial retinal OCT angiograms (45–47).

It has been well-documented that higher SUA levels may lead to hypertension (41). As a matter of fact, OCTA metrics may also be affected by hypertension (16) It is interesting to note that participants with higher SUA levels had more prevalent hypertension in our study. Findings from our studies may help explain why people with higher SUA levels are more likely to develop systemic microvascular disease (17, 18).

To our knowledge, this was the first study to identify a sex-specific association between SUA and RCP in a large community-based population using OCTA. The main strengths of this study include the use of detailed ophthalmic examination in a relatively large community-based study, adjustments for several potential confounding factors, a standardized quantitative system of retinal vascular plexus including magnification correction, and investigation using sensitivity analyses to ensure the robustness of the results.

However, several limitations in our study should also be discussed. First, we could not draw a causative association between SUA and RCP due to limitations of the cross-sectional nature of this study. There is no doubt that it is of great interest to explore the alterations of RCP in relation to high SUA levels over time, and it may be a future study objective as a follow-up to the JECS. Second, the study participants were all individuals from the Jidong community. This may not adequately represent other populations, and the applicability of our results to other racial/ethnic groups might be limited. More studies in other populations are required to further determine the effect of SUA on RCP. Finally, some potential confounders, such as hormone levels, cell factors that may affect SUA, retinal vasculature, and residual confounders, were not included in the analysis.

## Conclusion

In conclusion, we found that higher SUA levels were associated with lower superficial RCP vessel density in men. Given that the retinal microvasculature is an ideal window to observe the alternations of the microcirculation, our findings help validate the detrimental effect of high SUA levels on the retinal microvasculature and imply the importance of modulating SUA to prevent the microvascular alternation especially for men.

## Data Availability Statement

The raw data supporting the conclusions of this article will be made available by the authors, without undue reservation.

## Ethics Statement

The studies involving human participants were reviewed and approved by the Ethics Committee of the Staff Hospital of Jidong oil-field of Chinese National Petroleum. The patients/participants provided their written informed consent to participate in this study.

## Author Contributions

ML, LC, JQ, and FL designed and conceptualized the study and interpreted the data. KY analyzed the data. KY, XZ, YX, BS, CL, KS, and YJ had a major role in the acquisition of data. KY drafted the manuscript. ML, LC, JQ, and FL revised the manuscript for intellectual content. All authors read and approved the final manuscript.

## Funding

This study was supported by the National Natural Science Foundation of China, No. 81900903 (to ML); National Key R&D Program of China, No. 2020YFC2008200 (to FL) and 2019YFC0840708 (to LC); Zhejiang Provincial Natural Science Foundation of China, No. LY22H120007 (to LC); the Medical and Health Science and Technology Program of Zhejiang Province, No. 2017KY113 (to ML), No. 2018KY543 (to LC); Wenzhou Science and Technology Program, No. Y20210984 (to LC). The funding bodies played no role in the study design, collection, analysis, and interpretation of data.

## Conflict of Interest

The authors declare that the research was conducted in the absence of any commercial or financial relationships that could be construed as a potential conflict of interest.

## Publisher’s Note

All claims expressed in this article are solely those of the authors and do not necessarily represent those of their affiliated organizations, or those of the publisher, the editors and the reviewers. Any product that may be evaluated in this article, or claim that may be made by its manufacturer, is not guaranteed or endorsed by the publisher.

## Abbreviations

SUA, Serum uric acid; OCT, optical coherence tomography; OCTA, optical coherence tomography angiography; RCP, retinal capillary plexus; JECS, Jidong Eye Cohort Study; BCVA, low best corrected visual acuity; FBG, fasting blood glucose; LDL-C, low-density lipoprotein; HDL-C, high density lipoprotein; TC, total cholesterol; TG, triglyceride; eGFR, estimated glomerular filtration rate; AL, axial length; BMI, body mass index; CIs, confidence intervals.
